# The relationship between early and late event-related potentials and temperament in adolescents with and without ADHD

**DOI:** 10.1371/journal.pone.0180627

**Published:** 2017-07-25

**Authors:** Brittany R. Alperin, Hanna Gustafsson, Christiana Smith, Sarah L. Karalunas

**Affiliations:** 1 Department of Behavioral Neuroscience, Oregon Health and Science University, Portland, Oregon, United States of America; 2 Department of Psychiatry, Oregon Health and Science University, Portland, Oregon, United States of America; Vanderbilt University, UNITED STATES

## Abstract

Differences in emotional processing are prevalent in adolescents with attention deficit/hyperactivity disorder (ADHD) and are related to clinical impairment, but substantial heterogeneity exists. Within ADHD, some individuals experience difficulty with positive/approach emotions, negative/withdrawal emotions, or both. These problems may reflect differences in emotional reactivity, emotion regulation, or a combination, and the neurophysiological correlates remain unclear. Event-related potentials were collected from 109 adolescents (49 with ADHD) while they completed an emotional go/no-go task with three conditions: happy (positive/approach), fear (negative/withdrawal), and neutral. The P1 and N170 were used as a marker of early emotional processing and the P3b and late positive potential (LPP) were used as markers of later elaborative emotional processing. Emotional response style was assessed with parent and adolescent report on the Early Adolescent Temperament Questionnaire. There were no effects of emotion or group for the P1. Typically-developing adolescents exhibited a larger N170 to emotional vs. neutral faces while adolescents with ADHD showed the opposite pattern. All adolescents exhibited a larger P3b to fearful versus other faces and a larger LPP to emotional vs. non-emotional faces. Within the ADHD group, N170 responses to happy faces predicted parent ratings of positive/approach emotions. Findings highlight the importance of considering within-group heterogeneity when studying clinical populations and help clarify the time-locked neurophysiological correlates of emotion dysregulation.

## Introduction

Individuals who meet criteria for the same categorical DSM diagnosis may still vary widely in their symptoms presentations and other clinically-relevant traits. Attention-deficit/hyperactivity disorder (ADHD) is emblematic of the problems created by heterogeneity within psychiatric diagnostic categories [1]. Developmental instability and the lack of clear neurobiological bases for symptom-based presentations (e.g., inattentive, hyperactive, and combined presentations) hinder their use for either predicting disorder trajectory or treatment matching [2]. Thus, recent research guided by NIMH’s Research Domain Criteria (RDoC) initiative seeks to understand within-group variability by expanding beyond traditional symptom domains to consider other dimensions that may be relevant for refining psychiatric nosology to correspond to physiological mechanisms.

Although there are many dimensions of heterogeneity (e.g., neuropsychological profiles [3] and reward processing [4]), the importance of which will vary based on context and translational goals, the focus here is on emotion. Emotional response differences are prevalent in ADHD [5,6] and their presence is related to functional impairment [5,6] and risk for comorbid disorders [7,8]. Although the recent emphasis in the clinical literature has been on differences in negative emotions [9,10], emotional differences is not specific to negative valence contexts and can also manifest as differences in expression of positive emotions, such as excitement [5]. This is particularly the case in ADHD, where theories of the disorder have long pointed to upregulated positive/approach-motivation as one possible causal mechanism [11]. To this point, recent research has identified individual differences among individuals with ADHD in their valence-specific emotion response profiles based on temperament [12]. While some with ADHD show normative emotion profiles, others show high levels of either positive/approach emotions (i.e., “surgency”) or negative emotions (e.g., sadness, fear, anger). Preliminary data suggest that these emotion-based ADHD groups have distinct patterns of resting-state fMRI functional connectivity of the amygdala, suggesting a neurobiological basis for observed differences in emotional responding.

Temperament, as used here, describes a set of behavioral and incentive response tendencies, similar to personality traits in adults [13] that are related to information processing and emotional reactivity, regulation, and valence. At the broadest level, the differences in emotional expression captured by temperament measures reflects the combination of early automatic reactivity and later regulatory processing. Thus, consistent with recent neurobiological models of emotion dysregulation [14], an individual’s emotional expression reflects the interaction of early, physiological reactivity and later elaborative processing or regulation of this reactivity [13,15]. These early and later processes also interact [16,17], but the distinction provides a useful heuristic to describe the partially distinct processes of early and later stages of emotional processing.

Many theories have focused on ADHD as primarily a disorder of later, regulatory processing of emotion [18]; however, multiple pathway models suggest that early automatic processing may also play a role, particularly in the case of positive/approach emotions [19]. The current study further investigates this idea by examining the contribution of both early and late emotional processing to positive and negative affect in adolescents with ADHD. At the group level, individuals with ADHD show mixed evidence for differences in both early and later processing of emotional stimuli [10], and additional studies using time-locked central nervous-system measures are needed to clarify the neurobiological bases of emotional response differences in ADHD. The temporal precision of event-related potentials (ERPs) is ideal for clarifying the neurophysiological contributions to individual differences in emotion dysregulation. ERPs have a long history of application in studies of emotional processing and several components are sensitive to emotion [20,21] including: a) occipito-temporal P1 (peaking ~50–150 ms), b) occipito-temporal N170 (peaking ~150–200 ms) post-stimulus, c) centro-posterior P3b (~300–500 ms post-stimulus), and d) centro-posterior late positive potential (LPP; a slow wave extending beyond ~400 ms post-stimulus).

The current study focuses on the processing of emotional and non-emotional facial expressions. The ability to appropriately recognize and process emotional faces is particularly important for social interactions and generating the appropriate emotional responses in social contexts [22]. Those with ADHD are known to have difficulty with social interactions and are often rejected by their peers [23]. Although there seems to be a large body of evidence suggesting differences in facial emotion processing in ADHD [24,25], few studies have examined how the processing of emotional faces in ADHD changes across the processing stream [26,27]. Additionally, to the best of our knowledge, none have examined how early and late processing of emotional faces related to individual differences in emotional response style.

The current studies investigates both early and late processing of emotional faces. The P1 and N170 are used as markers of early emotional processing. Both of these components are involved in early perceptual processing of faces. The P1 is thought to be generated by the extrastriate cortex and reflects basic early visual processing of stimuli. Although not specifically sensitive to faces, the P1 has been found to be modulated by emotional face expression such that the P1 is larger to emotional vs. neutral faces, reflecting relative prioritization of emotional content [28]. The N170 has been specifically linked to the structural encoding of faces and is consistently larger to face than non-face objects [29,30]. A recent meta-analysis further confirms significant effects of emotion on the N170 with reliably larger amplitude N170 responses to angry, fearful, and happy expressions as compared to neutral expressions [31], and it has been examined as a marker of early emotional processing in several studies of ADHD in adults and adolescents [26,27,32,33].

The P3b and LPP are used as indicators of late or sustained emotional processing. P3b amplitude generally reflects the allocation of attention to task-relevant stimuli [34]. Emotional stimuli have been found to capture attention and are theorized to be automatically processed as task-relevant [21]. In line with this theory, a number of studies have found that the P3b is larger to emotional than non-emotional stimuli [35,36] including emotional vs. non-emotional faces [37–39]. More recently, researchers have noted increased positivities to emotional stimuli in a time window extending beyond the P3b [21]. In contrast to the P3b, which is responsive to a variety of manipulations including emotional salience, this later positive deflection, called the late positive potential (LPP), appears to be specifically sensitive to emotion with larger amplitude LPPs observed in response to both pleasant and unpleasant as compared to neutral stimuli, including emotional faces [40,41]. LPP amplitude is thought to reflect the degree of sustained, elaborative processing of emotional stimuli [42,43].

Within ADHD, patterns of findings for the P1 and N170 [26,27,32,33,44] or P3b and LPP components [26,27,44,45] are inconsistent, with studies variously reporting smaller, larger, and equivalent neurophysiological responses as compared to typically-developing individuals. Raz et al., (2015) found a larger P1 to emotional vs. non-emotional faces in adults with ADHD, as well as differences in the N170 component indicating individuals with ADHD responded more strongly to negative than positive affective faces. In contrast, Tye et al., (2014) and Ibáñez et al., (2014), who both focus only on the N170 component, found no differences between emotional and non-emotional faces for individuals with ADHD. Regarding later processing of emotional stimuli, Raz et al., (2015) found no differences in P3b emotion modulation between ADHD and control participants whereas Williams et al. (2008) found that those with ADHD generated smaller P3bs to anger and fear faces than controls and Singhal et al. (2012) found that those with ADHD have a larger LPP to fearful and sad faces than neutral.

Although there are relatively few studies of emotional face processing in ADHD, one intriguing possibility is that inconsistencies in findings may be attributable, in part, to emotional heterogeneity [12]. The current work tests this hypothesis directly by comparing P1, N170, P3b, and LPP responses under neutral, positive, and negative valence contexts between typically-developing adolescents and adolescents with ADHD, and then relating individual differences in neurophysiological responses to temperament ratings of both positive and negative affect. Typically-developing adolescents were expected to exhibit larger amplitude P1, N170, P3b, and LPP responses to emotional than non-emotional stimuli, whereas ADHD adolescents would not. However, this apparent weaker response to emotional stimuli in ADHD was hypothesized to be related to within-group heterogeneity, and neurophysiological responses within the ADHD group were expected to predict individual differences in emotional response style.

## Methods

### Participants and recruitment

109 individuals (n_ADHD_ = 49) were recruited from a larger, ongoing longitudinal study and were invited to participate in an additional EEG testing visit. Recruitment for the larger study uses a community-based strategy based on public advertising and outreach. A parent/legal guardian provided written informed consent, and adolescents provided written assent for the study. Ethics approval was obtained from the Institutional Review Boards at Oregon Health & Science University.

After an initial screening phone call, a parent/guardian and teacher completed standardized rating scales, including the Conners’ Rating Scales, 3^rd^ edition [46] and the ADHD Rating Scale (ADHD-RS) [47]. The parent/guardian also completed a semi-structured clinical interview (Kiddie Schedule for Affective Disorders and Schizophrenia, K-SADS) administered by a Master’s-level clinician who had achieved research reliability [48]. The parent/guardian reported on lifetime and current symptom levels, as well as age of onset and impairment. IQ was estimated based on a reliable and valid two-subtest short form of the WISC-IV (Block Design and Vocabulary; [49]).

Final DSM-IV diagnostic groups were determined as follows: individuals with ADHD were required to meet full diagnostic criteria for ADHD on the K-SADS, including symptom counts, impairment, and age of onset. Although cross-situational severity is incorporated into the K-SADS diagnosis, to confirm this we also required children in the ADHD group to have at least one parent *and* one teacher rating of inattention and/or hyperactivity that exceeded 85^th^ percentile (T-score = 60). Those in the typically-developing control group were required to have no parent or teacher ratings exceeding T-score = 60 *and* to not meet diagnostic criteria for ADHD on the K-SADS. Further, to avoid inclusion of borderline cases in the typically-developing group, these adolescents were required to have 3 or fewer current hyperactivity symptoms, 3 or fewer current inattention symptoms, *and* 4 or fewer total symptoms. Total symptom counts were determined by combining parent (K-SADS) and teacher (ADHD-RS) report using an “OR” algorithm [50].

### Exclusion criteria

Adolescents were excluded from the current study if they were prescribed long-acting, non-stimulant psychotropic medications; had self-reported history of neurological impairment such as seizures or head injury with loss of consciousness; had a history of substance abuse; had prior diagnosis of intellectual disability, autism spectrum disorder, or psychosis; were currently experiencing a major depressive episode; or had estimated IQ < 70.

### Medication washout

Adolescents with ADHD taking stimulant medications were included in the study but were required to be off medication for 24 (for short-acting preparations) to 48 hours (for long-acting preparations) prior to testing.

### Temperament ratings

A parent/guardian and the adolescent separately completed the Early Adolescent Temperament Questionnaire (EATQ; [51]), which is derived from a well-regarded conceptual model using confirmatory factor analysis [52]. The *surgency* factor and fear subscale were used in the current study. Surgency is related to extraversion and experience of positive emotions (e.g., excitement), and is associated with approach tendencies [11,15]. The fear subscale was selected as a measure of withdrawal-related negative affect. Parent-reported surgency and adolescent-reported fear are used in all analyses consistent with literature suggesting that in adolescence, self-report of internal states and parent-report of observable behaviors are most reliable [53]. 5.8% of parent reported and 11.8% of adolescent reported temperament data was missing due to incomplete questionnaires and were handled using missing data procedures described below.

### Experimental procedure

**Fig 1:**
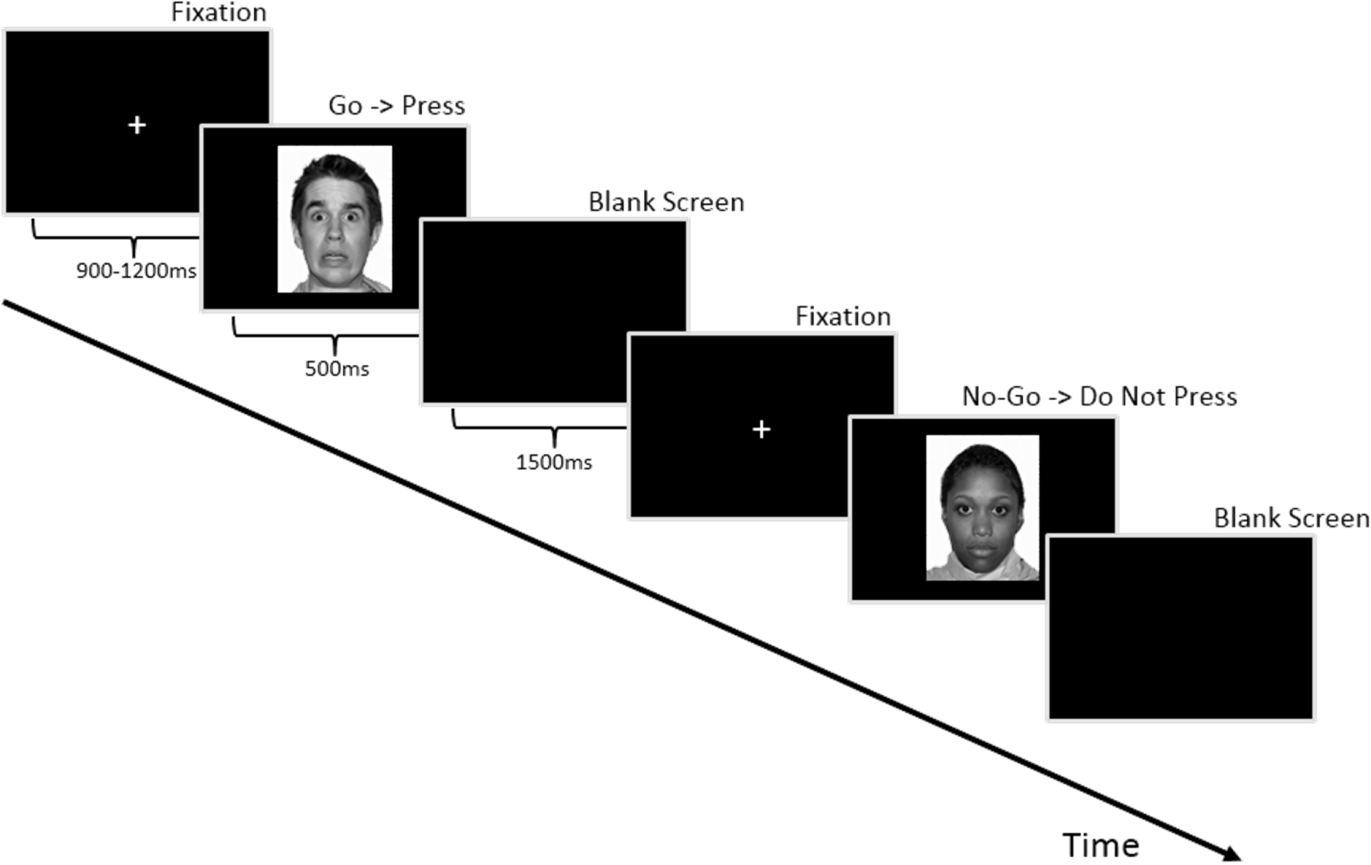
Depiction of an example experimental run.

**Fig 1**depicts an example experimental run. Participants completed three separate conditions (fear, happy, neutral) of an emotional go/no-go task (70% go, 30% no-go) in counterbalanced order. For each condition, participants were shown a series of grey-scale faces from 12 individuals (6 female) from the NimStim set [54] and were asked to respond by button press to specified target faces. Within each condition, 172 trials were presented in semi-random order in two equal blocks (total of 344 trials). In the emotionally-salient (fear or happy) conditions, participants responded with a key press to emotional expressions and withheld responding to faces with neutral expressions. In the neutral condition, all faces had neutral expressions and participants responded to either male or female faces and withheld responding to the other sex (counterbalanced by block). Analyses here focus on the go trials because these are the stimuli that contain emotional content and our primary hypotheses here were related to processing of emotional stimuli. This approach is consistent with other studies, which similarly uses cognitive paradigms (e.g., oddball or go/no-go tasks) [26,27,33,55,56], but focus on response to specific subsets of stimuli. Although we do not consider no-go trials here, the use of a task that demands attention and response to specific stimulus types may be particularly important for eliciting effects for emotional face stimuli in clinical groups that are not seen in other types of tasks [57].

### ERP recordings

EEG was recorded at 500Hz with 32 Ag-AgCl active electrodes using PyCorder v1.0.9. The electrode array was based on the international 10–20 system centered at Cz. EEG signals were amplified by a BrainVision actiCHamp2 amplifier (Cary, NC). Recordings were referenced to Cz online then re-referenced offline.

### Data analysis

EEG data were analyzed using ERPLAB [58] and EEGLAB [59] toolboxes for MATLAB. Raw EEG data were resampled to 250 Hz and referenced offline to the average of all channels. EEG signals were filtered using an IIR filter with a bandwidth of .01–50 Hz [60]. Eye artifacts were removed through independent component analysis.

Epochs from -200 to 1000 ms were time-locked to the onset of the face stimuli. A 200 ms prestimulus period was used for baseline correction. Trials were discarded from the analyses if they contained baseline drift or movement artifacts greater than 90 μV. Only correct responses to go trials in each of the three conditions (fear, happy, neutral) were included in analyses because the emphasis of the current work was on responses to the emotional faces, specifically. Based on *a priori* criteria, a participant’s data for a given condition were excluded from analyses if >50% of the total trials were rejected due to artifacts. This resulted in exclusion of 1.8% of data (1 fear, 2 neutral, and 3 happy condition). Missing data were handled via missing data methods described below.

Component amplitudes were measured as mean area amplitude in a specified stimulus-locked time windows and at electrode sites selected based on those used most commonly in the literature [60]: a) *P1*: between 60 and 160 ms post stimulus at bilateral occipito-temporal electrode sites P7 and P8; b) *N170*: between 150 and 250 ms post stimulus at the bilateral occipito-temporal electrode sites P7 and P8; c) *P3b*: between 300 and 600 ms post stimulus at centro-posterior electrode sites P3, Pz, and P4 [34]; and d) LPP: between 600 and 1000 ms post stimulus also at centro-posterior electrode sites P3, Pz, and P4 [21].

### Statistical analysis

Cognitive performance and ERPs were analyzed separately using a linear mixed model with a compound symmetry repeated covariance structure in SPSS (v.22.0). Linear mixed models are a well-established method for analyzing data with repeated measures which, in contrast to methods like repeated measures ANOVA, has the advantage of being able to accommodate missing dating [61–63]. The linear mixed effect model specified in the current analysis is nearly identical to a repeated measures ANOVA; the main difference is that in these analyses missing data are handled using maximum likelihood estimation rather than listwise deletion [64]. For regression analyses, ERP difference waves were calculated by subtracting the mean amplitude from the neutral condition from that of the emotional conditions (i.e., fear-neutral and happy-neutral). Relationships between temperament (surgency or fear) and ERP difference waves were examined using moderation analyses in MPLUS (7.2). Predictors (ERP difference waves) were mean centered prior to model parametrization. Age and sex were used as covariates in all analyses. Although IQ differed between groups, this was not used as a covariate since a lower IQ often accompanies ADHD and by controlling for this variable we may be controlling for a feature inherent to the disorder [65]. The number of trials was also used as a covariate in linear mixed models including ERPs. Missing data were handled using maximum likelihood methods [66]. A false discovery rate (FDR) correction for multiple comparisons was used on all analyses with a false discovery rate set at .05. FDR corrected p-values are presented for all tests.

## Results

### Sample description & task performance

See **Table 1**for participant demographic, clinical, and cognitive performance scores. As expected, for go accuracy, there was a main effect of group (*F*(1,109.3) = 10.04, *p* = .002, *d* = .60), such that accuracy was higher for controls than for ADHD participants. There was also an effect of condition (*F*(2,217.5) = 9.15, *p* < .001) such that go accuracy was lower during the fear condition than the other two (fear < neutral = happy). There was no group x condition interaction (*F*(2,217.5) = 2.18, *p* = .26).

**Table 1:** demographic information and clinical and cognitive performance scores (means(SD)).

	*Control*	*ADHD*	*F*	*Effect Size (95% CI)*
N	60	49		
Age (years)	13.87 (1.08)	13.70 (1.48)	0.48	0.13 (-.32-.66)
IQ	116.23 (11.72)	107.24 (14.92)	12.41**	0.67 (3.93–14.05)
(Boys:Girls)	38:22	42:7	*X*^2^ = 6.92*	
*ADHD presentation type (%)*				
Inattentive	—	61.90		
Hyperactive	—	4.76		
Combined	—	33.33		
*EATQ*				
Surgency^a^	3.60 (0.58)	3.50 (0.62)	0.59	0.17 (-.13-.34)
Fear^b^	2.41 (0.63)	2.58 (0.80)	1.36	0.23 (-.46-.12)
*Conners’ (Parent)*				
Hyperactivity	48.55 (9.44)	70.60 (15.52)	78.43**	1.72 (17.11–26.98)
Inattention	48.23 (10.67)	75.26 (10.63)	164.65**	2.53 (22.85–31.20)
Learning Problems	46.23 (4.78)	61.87 (11.78)	81.55**	1.74 (12.21–19.08)
Executive Functioning	49.20 (10.00)	70.79 (11.90)	100.20**	1.96 (17.31–25.87)
*Conners’ (Teacher)*				
Hyperactivity	48.83 (7.34)	62.32 (14.58)	31.21**	1.17 (8.69–18.29)
Inattention	48.62 (7.53)	65.32 (12.10)	62.05**	1.66 (12.49–20.91)
Learning Problems &	47.62 (6.89)	58.56 (10.97)	32.24**	1.19 (7.11–14.78)
Executive Functioning				
*Go Accuracy (%)*^*c*^			10.04*	0.60 (.02-.07)
Fear	96.10 (9.30)	89.60 (9.10)		
Neutral	97.30 (9.30)	93.90 (9.10)		
Happy	98.40(9.30)	95.90 (9.10)		
*RT (ms)*^*c*^			0.08	0.06 (-36.25–48.22)
Fear	573.29 (123.99)	557.58 (124.40)		
Neutral	526.01 (123.99)	526.18 (124.40)		
Happy	503.90 (124.55)	501.49 (124.40)		

Significance based on FDR corrected *p* values. EATQ = Early Adolescent Temperament Questionnaire; Connors’ = Connors’ rating scale; RT = reaction time

**p < .001

*p < .01

^a^ parent report

^b^ child report

^c^ estimated marginal means after controlling for age and sex presented

For go RTs, there was no effect of group (*F*(1,109) = .08, *p* = .80, *d* = .08). However, there was a main effect of condition (*F*(2,217.1) = 19.89, *p* < .001) such that RTs were faster in the happy condition than the neutral conditions, consistent with greater approach-motivation, and slower in the fear condition than the neutral condition, consistent with greater withdrawal. The group x condition interaction was not significant (*F*(2,217.1) = .36, *p* = .79). Overall, the behavioral performance measures suggest that both groups responded as expected to the emotional manipulation.

### ERPs

**Fig 2:**
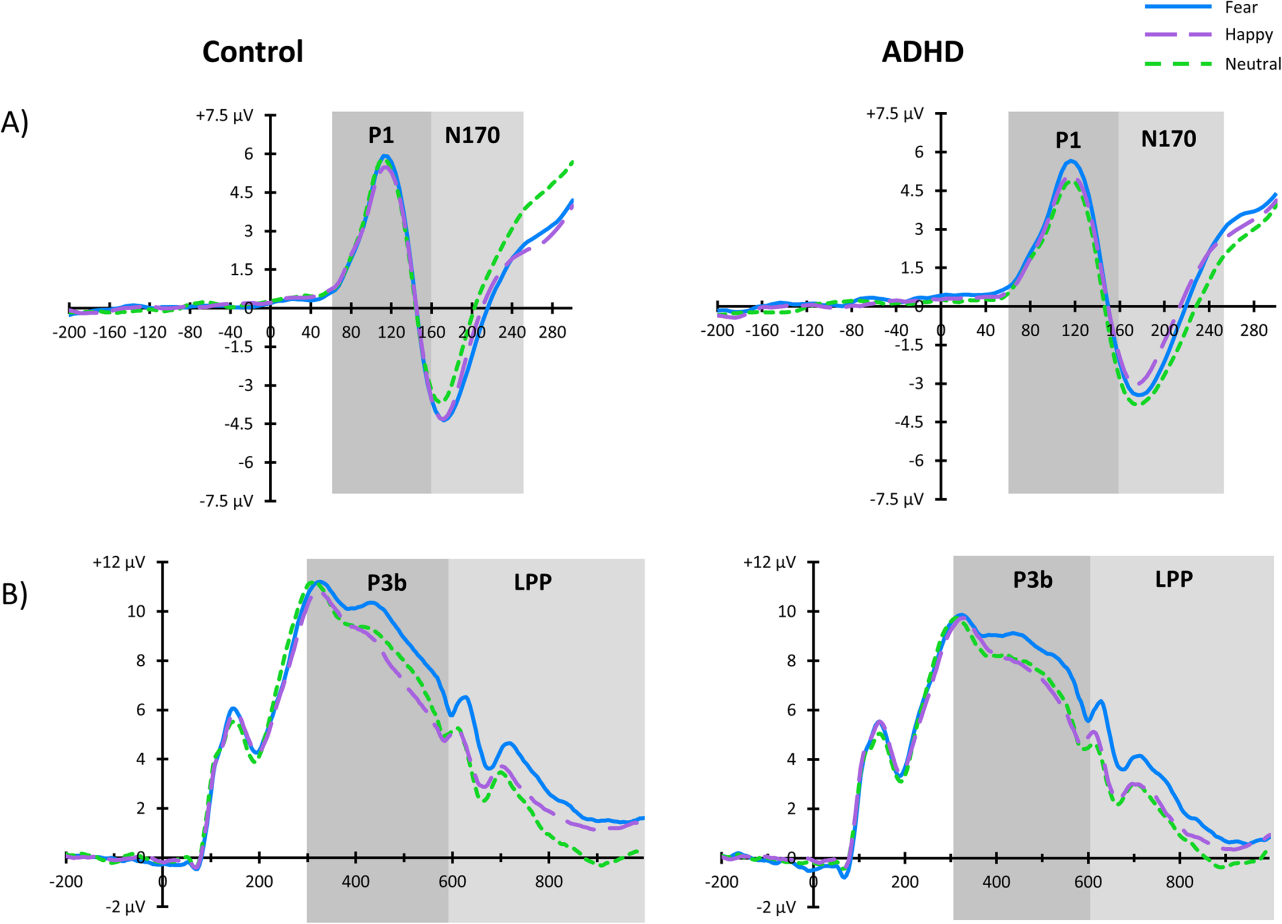
Illustration of the grand average ERP waveforms for the A) N170 at electrode site P7 and P8 collapsed, and B) P3b and LPP at electrode sites P3, P4, and Pz collapsed.

**Fig 3:**
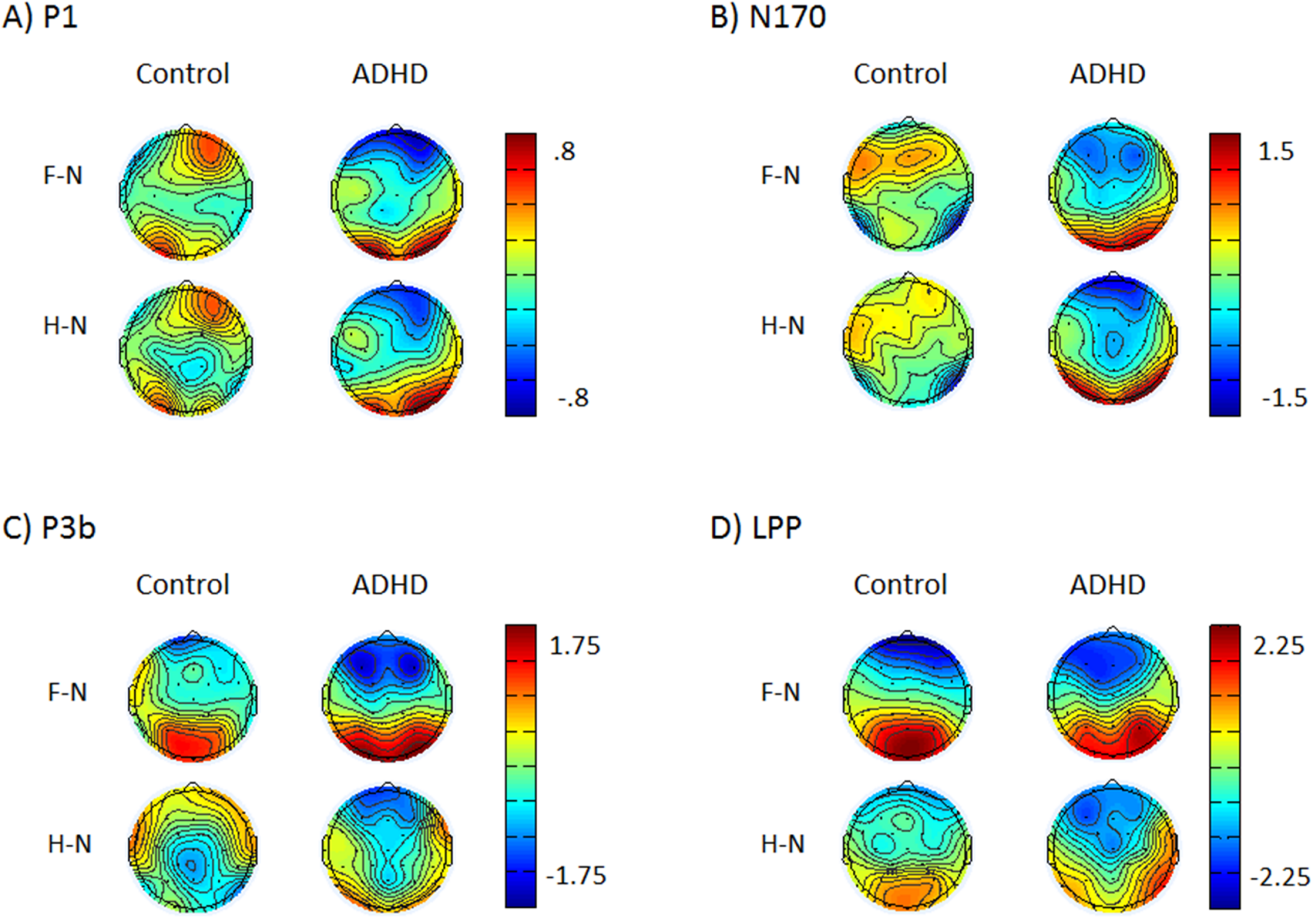
Topographic head plots of the fear-neutral (F-N) and happy-neutral (H-N) difference waves for the A) P1, B) N170, C) P3b, and D) LPP.

**Fig 2**depicts the grand average waveforms and **Fig 3**depicts topographic head plots of the difference waves for each component.

#### P1

There was no main effect of group (F(1,104) = .91, p = .51, *d =* .05) or condition (F(2,520) = 1.12, p = .51). There was a main effect of electrode (F(1,520) = 20.04, p <. .001, *d* = .47) such that the P1 was largest at electrode site P8. There was no group x condition interaction (F(2,520) = 1.97, p = .30) or any interactions with electrode (all ps > .51).

#### N170

There was no main effect of group (*F*(1,104) = .15, *p* = .79, *d* = .07), condition (*F*(2,520) = .1.02, *p* = .51), or electrode (F(1,520) = 1.31, p = .45, *d* = .10). However, lack of main effects was qualified by a significant group x condition interaction (*F*(2,520) = 8.08, *p* = .001). Adolescents with ADHD had a larger N170 to non-emotional that to emotional faces (neutral > fear = happy), while typically-developing adolescents showed the opposite pattern with larger N170 to emotional than non-emotional faces (fear = happy > neutral). There was also a group x electrode interaction (F(1,520) = 5.98, *p* < .05) such that the control group had a larger N170 response at electrode site P7 whereas the ADHD group demonstrated a similar amplitude response at all sites. There were no other interactions with electrode site (all *p*s > .79)

#### P3b

There was no main effect of group (*F*(1,104) = 1.80, *p* = .36, *d* = .26). There was a main effect of condition (*F*(2,832) = 23.31, *p* < .001) such that the P3b response under the fear condition was larger than under the neutral or happy conditions (fear > neutral = happy). There was also a main effect of electrode (*F*(2,832) = 37.08, *p* < .001) due to the P3b at electrode site P3 being smaller than at the other two electrode sites (P3 < P4 = Pz). There was no condition x group interaction (*F*(2,832) = .26, *p* = .80) or any interactions with electrode (all ps > .07).

#### LPP

There was no main effect of group (*F*(1,104) = .85, *p* = .51, *d* = .18). There was a main effect of condition (*F*(2,832) = 50.12, *p* < .001) such that the LPP was larger under the fear condition followed by the happy condition and finally the neutral condition (fear > happy > neutral). There was a main effect of electrode (F2,832) = 130.56, p < .001) such that the LPP was largest at electrode site Pz followed by P4 and smallest at P3 (Pz > P4 > P3). There was no condition x group interaction (*F*(2,832) = 1.67, *p* = .36) or any other interactions with electrode (all ps > .05).

### Relationship between ERPs and temperament

We next examined relationships between neurophysiological response and emotional response style by diagnostic group. Because there were no electrode x condition or group x electrode x condition effects, moderations were run for each component averaged across electrode sites (i.e., P7/P8 for the P1 and N170 and P3/P4/Pz for the LPP and P3b). However, for completeness, we also note effects at each site separately. Beta weights and standard errors for primary regressions and moderation analyses are presented in **Table 2**.

**Table 2:** Regression coefficients.

	Surgency		Fear	
Variable	β	SE	β	SE
**P1 H-N**	.12	.10	—	—
**P1 H-N group interaction**	-.05	.72	—	—
**P1 F-N**	—	—	-.001	.12
**P1 F-N group interaction**	—	—	-.09	.17
**N170 H-N**	-.15	.14	—	—
**N170 H-N x group interaction**	-.34*	.13	—	—
**N170 F-N**	—	—	-.02	.88
**N170 F-N x group interaction**	—	—	.10	.12
**P3b H-N**	-.10	.11	—	—
**P3b H-N x group interaction**	.04	.17	—	—
**P3b F-N**	—	—	.12	.11
**P3b F-N x group interaction**	—	—	.09	.17
**LPP H-N**	-.10	.09	—	—
**LPP H-N x group interaction**	.23	.14	—	—
**LPP F-N**	—	—	.08	.13
**LPP F-N x group interaction**	—	—	-.09	.23

Standardized coefficients presented. Significance level based on FDR corrected *p* values. F-N = fear–neutral; H-N = happy = neutral.

* *p* < .05

#### Surgency

**Fig 4:**
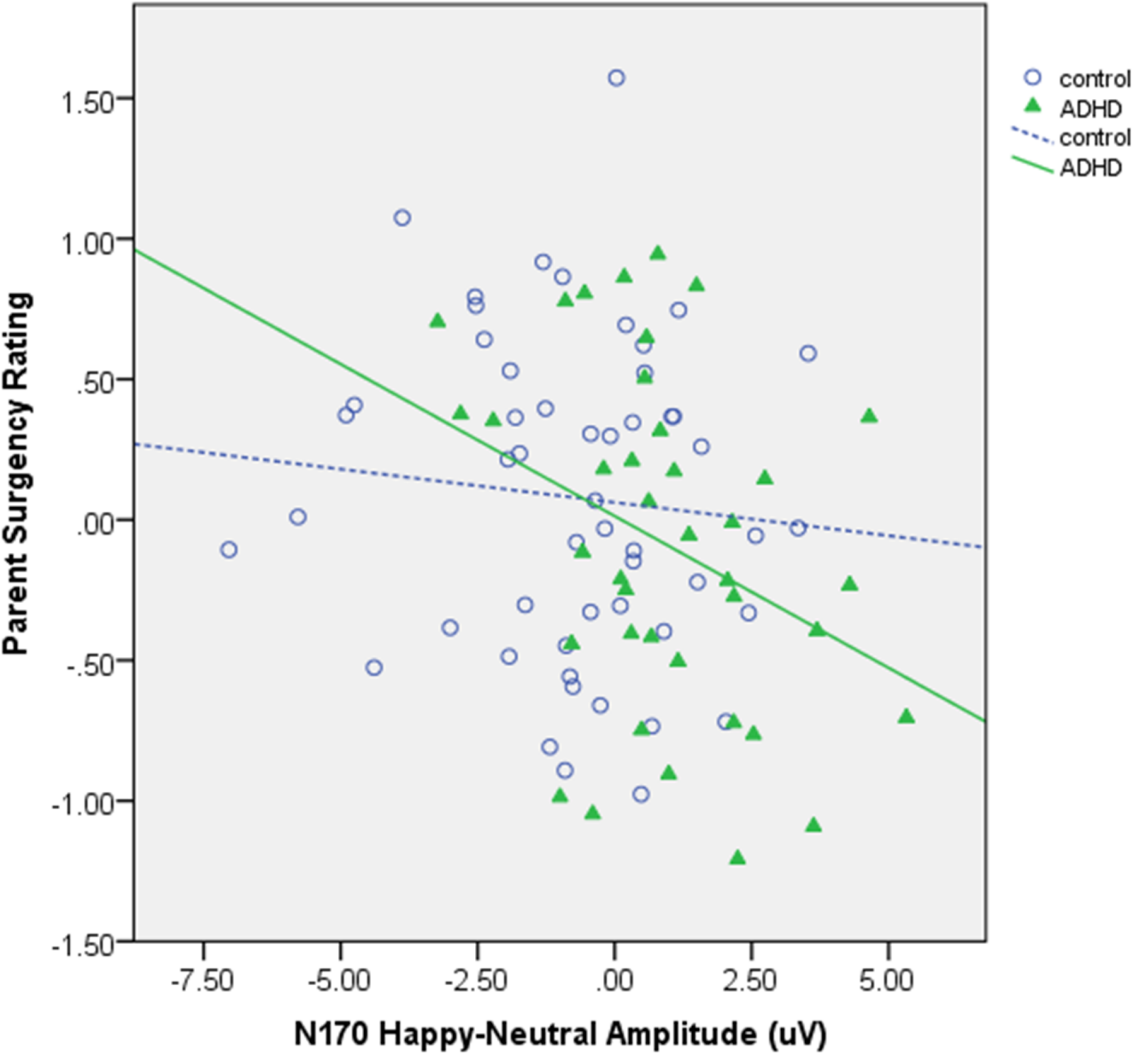
Scatterplots of significant regressions between N170 happy-neutral difference wave and surgency. Unstandardized residuals of temperament ratings are represented.

As shown in **Table 2**and **Fig 4**, group significantly moderated the relationship between N170 amplitude and surgency, *β* = -.34, *p* = .04. A larger N170 in the happy condition, indicating greater early response, was related to higher surgency within the ADHD group, *β* = -.43, *p* < .001, but not the control group, *β* = .01, *p* = .94. This pattern was significant at electrode site P8 (p = .008), but not at P7 (p = .17).

There were no significant moderation effects between a surgent temperament and the P1, P3b, or LPP in the primary analyses (all ps > .20). However, secondary analyses examining effects at each electrode site found a significant moderation by group of the relationship between LPP and surgency at site Pz (where LPP was largest for both groups; *β* = -.30, *p* = .02. Results indicated that a smaller LPP was related to greater surgency in the control group (p = .001), but not the ADHD group (p = .80). This effect was not significant at lateral electrode sites P3 and P4.

#### Fear

There were no significant moderation effects between a fearful temperament and any of the ERP measures (all ps > .40). See **Table 2**.

## Discussion

Emotional response is a critical dimension of heterogeneity in ADHD, but the neurophysiological correlates of these differences and the contributions of relatively earlier and later processing are not clear. In the current study, typically-developing adolescents showed expected patterns of neural differentiation between emotional and neutral faces for the N170, while those with ADHD did not. If not explored further, group-level results suggest that adolescents with ADHD have a blunted early neurophysiological response to emotional stimuli. In contrast, further examination demonstrated that magnitude of the N170 response in the ADHD group to positive emotional stimuli was related to emotional heterogeneity, with larger N170 response predicting higher surgency. These findings are in line with prior theory suggesting that excessive approach-motivation and response to positive incentives in ADHD is not driven solely by regulatory deficits, but can also be related to differences in earlier, potentially more reactive processing in the absence of differences in later regulatory processing [19].

Interestingly, the relationship between early emotional processing and temperament were only present for the N170 and not the P1. In the current study, the P1 was not modulated by emotional expression. The N170, which is specifically related to encoding of facial features, and was larger to both fearful and happy expressions in our typically-developing sample, may be more sensitive to individual differences in emotional face processing for both negative and positive expressions, particularly as these difference relate to emotional response style. Future work should continue to investigate the functional differences between the P1 and N170 in terms of emotional processing and how these processes related to real-world functioning.

Most studies of emotional responding to-date have used the P3b as a marker of later emotional processing of stimuli. The P3b component is not specifically sensitive to emotion and reflects other processes, such as working memory updating and decision making [67]. Although both the P3b and LPP have been found to be sensitive to emotion, in the current sample the P3b was only larger under the fear condition, whereas responses to happy and neutral stimuli did not differ. This may be consistent with threat-related information being more highly prioritized for processing than other emotional information and, therefore, more likely to be reflected in ERP components that are broadly related to attention and cognition. In contrast, the LPP was larger under both the fear and happy conditions as compared to the neutral condition. Given its unique association with emotional processing, the LPP may be a more sensitive measure of later, elaborative emotional processing than the P3b and/or may be sensitive to a broader range of emotions. However, these two components spatially and temporally overlap [20,21]. Future work in our lab will continue to investigate the relationship between the P3b, LPP, and emotion regulation in ADHD.

Of note, our primary analyses indicated no relationship between the P3b or LPP and individuals’ emotional response styles, despite the fact that these components differed between emotion conditions. We also failed to identify any relationships between ERP components (early or late) and fearful temperament. Face stimuli are commonly used in studies of emotional processing and elicit emotional responses [31]. In our study, in addition to emotional effects on ERP components, reaction times differed in the expected directions by condition for both groups, giving us confidence that the emotional manipulation was effective. Nonetheless, emotional scenes may elicit a more robust neurophysiological responses than faces. Future work examining the relationship between ERPs (particularly later components) and negative affect using a variety of emotional stimuli types may be informative.

One caveat to the lack of relationships between later ERP components and emotional response style was a secondary finding that a smaller LPP in the happy condition predicted higher surgency in typically-developing controls. However, this effect was only significant at electrode site Pz and not in the primary analyses which collapsed across electrode sites. Although there was no condition x electrode interaction, the LPP response was largest at Pz. If replicated, this finding may suggest partially distinct neurophysiological correlates for increased positive approach-motivation in normative adolescent development and ADHD. This is an intriguing possibility, but should not be over-interpreted given the secondary nature of the analyses by electrode site. Additional studies that take into account LPP topographical difference in those with and without ADHD are needed.

As expected [68], ADHD participants generally performed more poorly than non-ADHD controls on the go/no-go task. The current study focuses on relationships between emotional face processing and emotional response styles in ADHD, and therefore analyses were restricted to the emotional go stimuli. Emotional effects on inhibitory control and other measures of executive function remain relatively poorly characterized [69,70], and additional studies of how the inhibitory process is affected by emotional context, although outside the scope of the current work, will be important. This is a direction for our own future work and will be important for integrating across the multiple domains of heterogeneity that likely play a role in ADHD and other clinical disorders.

The groups in the current study were not matched for sex with proportionally more males in the ADHD than control group, consistent with well-documented sex differences in rates of ADHD diagnoses [71]. Sex was used as a covariate in all analyses, suggesting current findings are not attributable to sex differences in the samples. However, future research focused specifically on the effects of sex on the relationship between emotional response style and ERP markers of emotional processing in adolescents with ADHD will be important.

### Summary

The current study identifies neurophysiological correlates of emotional heterogeneity in ADHD. Results are consistent with prior theories identifying over-reactivity to positive affective cues, even in the presence of normative regulation, as one pathway leading to ADHD impairment. Parsing this emotional heterogeneity will be important for RDoC-informed efforts to identify more neurobiologically-informed subgroups within ADHD and other clinical populations.

## Supporting information

S1 Table The dataset used in the current study. (XLSX)
